# Population screening for TAVI procedure

**DOI:** 10.1186/1749-8090-8-S1-O46

**Published:** 2013-09-11

**Authors:** A Redzek, L Velicki, M Petrovic, B Mihajlovic, M Fabri, S Nicin

**Affiliations:** 1 Cardiovascular Surgery, Institute of Cardiovascular Diseases Vojvodina, Serbia; 2 Cardiology, Institute of Cardiovascular Diseases Vojvodina, Serbia

## Background

Transcatheter aortic valve implantation (TAVI) is an alternative that offers hope to patients who are older, or have contraindication to conventional surgical aortic valve replacement due to considerable comorbidities. In our country there is about 500 to 1000 patients, older than 80 years needing this, less invasive procedure. The aim of this study was to asses the number of high-risk patients with severe aortic stenosis, hospitalized at our institute, which are candidates for percutaneous aortic valve implantation.

## Methods

We have prospectively analyzed all patients who were hospitalized at our institute, between April 2011 and November 2012, due to severe aortic stenosis. Each of these patients was discussed in relation to inclusive criteria for TAVI.

## Results

In the period of 20 months, there were 374 patients hospitalized due to severe aortic stenosis at our institute. In the group of patients older than 80 years (13.6%; 51/374), low ejection fraction, pulmonary hypertension, significant carotid stenosis, atrial fibrillation and significant mitral regurgitation were registered in 5.9%, 29.4%, 13.7% , 21.6% and 25.5% respectively. On the other hand, percentage of surgicaly treated patients with severe aortic stenosis decreases in older gruops of patients, especially in the age over 80 years (90.2%). Most of patients from this subgroup were treated conservatively due to high operative risk for conventional surgerical procedure.

## Conclusion

The data obtained in this study indicate a high percentage of conservatively treated patients with severe aortic stenosis and high operative risk in patients over 80 years. This data confirm needs for implementation of TAVI in our population.

